# Ellis-Van Creveld Syndrome: A Rare Case Report of an Indian Child With Rare Cardiac Anomalies and Normal Intelligence

**DOI:** 10.7759/cureus.29846

**Published:** 2022-10-02

**Authors:** Benumadhab Ghosh, Isha Sahai, Gajendra Agrawal, Sourya Acharya, Johann Christopher

**Affiliations:** 1 Department of Cardiology, Jawaharlal Nehru Medical College, Datta Meghe Institute of Medical Sciences, Wardha, IND; 2 Department of Medicine, Jawaharlal Nehru Medical College, Datta Meghe Institute of Medical Sciences, Wardha, IND; 3 Department of Cardiology, CARE Hospital, Hyderabad, IND

**Keywords:** ellis-van creveld syndrome, vsd, asd, clubbing, tachycardia, tachypnea, polydactyly

## Abstract

Ellis-Van Creveld syndrome (EVCS) is an abnormal genetic condition of the *EVC2* gene located on chromosome 4. In this case, the person presents with bone growth abnormalities, thus having a short stature, short arms and legs (more commonly the forearm and lower leg), a narrow chest with short ribs, polydactyly, spoon-shaped or malformed nails, abnormalities in dentition, and congenital heart defects like atrial septal defects and ventricular septal defects. In this case report, we present a 4.5-year-old female child who presented with cough and cyanosis as signs and tachypnea, tachycardia, facial oedema, cold, and clubbing as symptoms with polydactyly and short stature focuses on a rare presentation of a syndromic disease known as EVCS.

## Introduction

Ellis-Van Creveld syndrome (EVCS) is also called chondroectodermal dysplasia. It is a rare genetic abnormality that is autosomal recessive in nature. The *EVC* gene is located in chromosome 4p16 which is plotted in the short arm of chromosome 4 [1,2,3]. This syndrome is due to the mutation within a non-homologous gene called the *EVC2* gene. This *EVC2* gene is located close to the *EVC* gene following a head-to-head configuration [4]. Simon van Creveld and Richard Ellis described the term first in the 1940s, naming it chondroectodermal dysplasia [5]. The prevalence rate of EVCS seen in the Amish population of Pennsylvania, USA was one out of 5,000 total live births; whereas in the non-Amish population, the prevalence rate was found to be seven out of 1,000,000 [6]. The important features of this rare syndrome are polydactyly, chondroectodermal dysplasia, and congenital heart defects like defects in the atrial septum, ventricular septum, and patent ductus arteriosus.

*EVC2* and *EVC* are important in skeletal and endochondral development and also code for ciliary basal body proteins. The patients have inordinate small statures like in acromesomelic dwarfism, short limbs and extraordinarily long trunk, fine sparse hair, and hypoplastic nails of a finger. There are various manifestations in the oral region [7,8]. They include several muscular fibrous frenulums, dental transposition and conical appearance of teeth, hypodontia (less number of teeth), and hypoplasia of the teeth enamel; imperfect positioning of the teeth is observed when jaws are shut (malocclusion). Premature eruptions and exfoliation are seen in these patients' teeth [1-3,5,9]. In their work, Gorlin et al. have observed that patients who have survived infancy have a life expectancy like ordinary people. There is a record of the oldest living patient aged 82 years [10].

In this case report, we are describing a clinical case scenario of a 4.5-year-old female patient who visited the paediatric cardiology department of the Acharya Vinoba Bhave Rural Hospital (AVBRH), Wardha, India. She presented with distinctive clinical features suggestive of EVCS with three pulmonary veins, which is rare, along with other rare cardiac anomalies and normal intelligence.

## Case presentation

A child aged 4.5 years was brought by her parents with the chief complaints of fascial oedema which progressed to anasarca over two days. There was a marked increase in the effort needed to breathe along with an increased respiratory rate. The patient had no history of associated fever, chest pain, or forehead sweating but was suffering from severe cough and cold The patient had a similar history of complaints a few months back and got treated with intravenous antibiotic injections. Our patient had a history of recurrent respiratory tract infections. 

**Figure 1:**
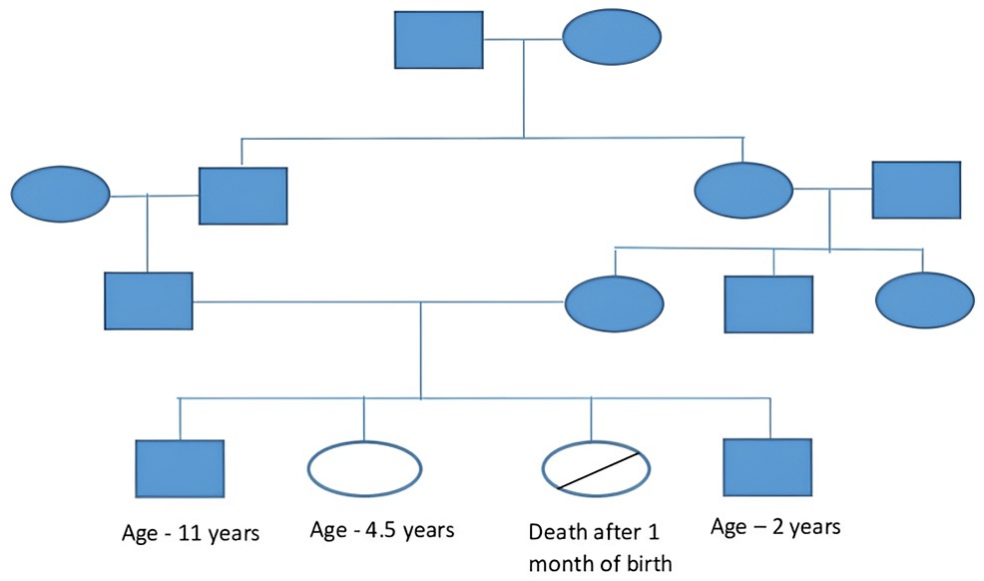
Family tree of the patient

Family history as stated by her mother has been explained in which a female sibling with similar clinical conditions died after one month of her life. A male older sibling and a male child born after the third child to the parents were normal. There was no significant family history in earlier generations showing similar clinical conditions as seen in Figure 1.

At the time of birth, the neonate weighed around 2.5 kg. There was no history of neonatal intensive care unit (NICU) stay. The patient's mother confirmed that there was delayed development of the patient in regard to physical growth. The motor developmental milestone was delayed (she started walking late). She has not undergone any physical therapy and did not follow up regarding the same. The family was not aware of the patient's clinical conditions until she visited our hospital. She has been immunized as per National Immunization Schedule. Anthropometric history shows severe stunting (as per WHO standards) and grade 2 malnourishment (as per the Indian Academy of Pediatrics). Her measurements are listed in Table 1.

**Table 1:** Anthropometric measurements of the patient

	OBSERVED	EXPECTED	PERCENTAGE
HEIGHT/LENGTH	90 cm	107 cm	84%
WEIGHT	11 kg	17 kg	64%
HEAD CIRCUMFERENCE	47 cm	49.5 cm	94%

**Figure 2:**
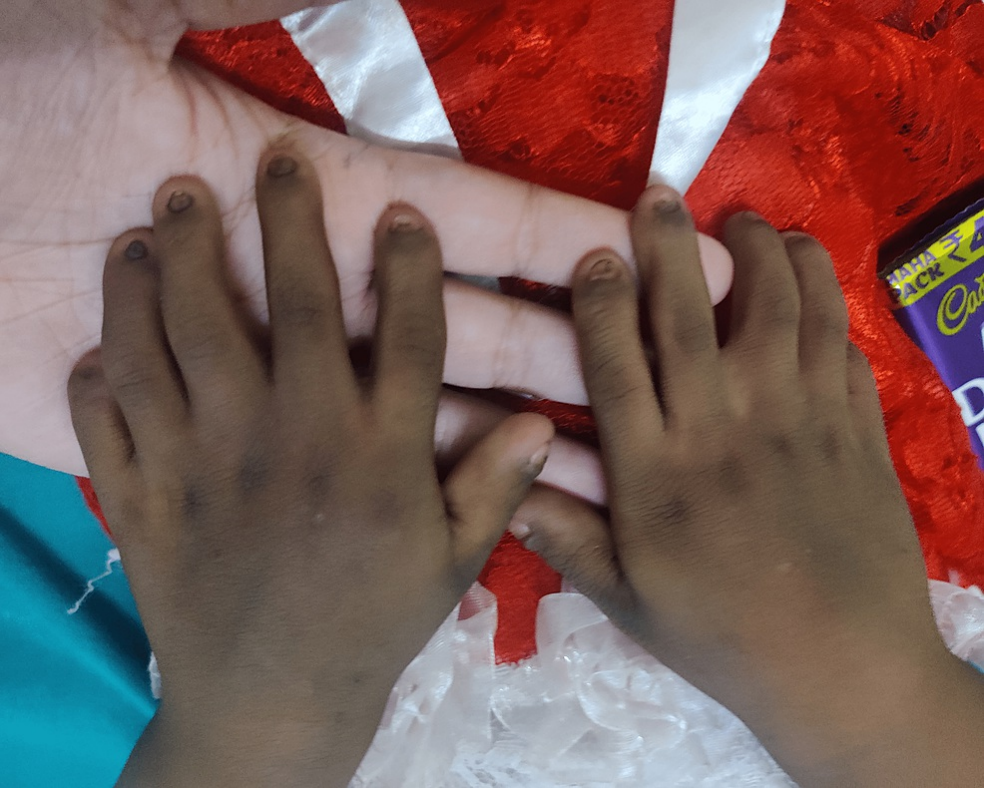
Showing polydactyly

**Figure 3:**
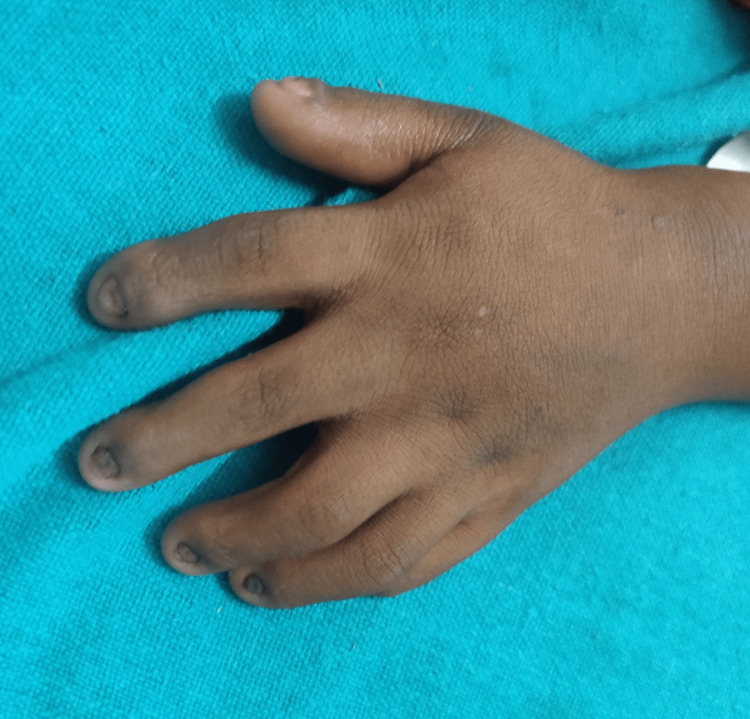
Showing fingernail dysplasia

**Figure 4:**
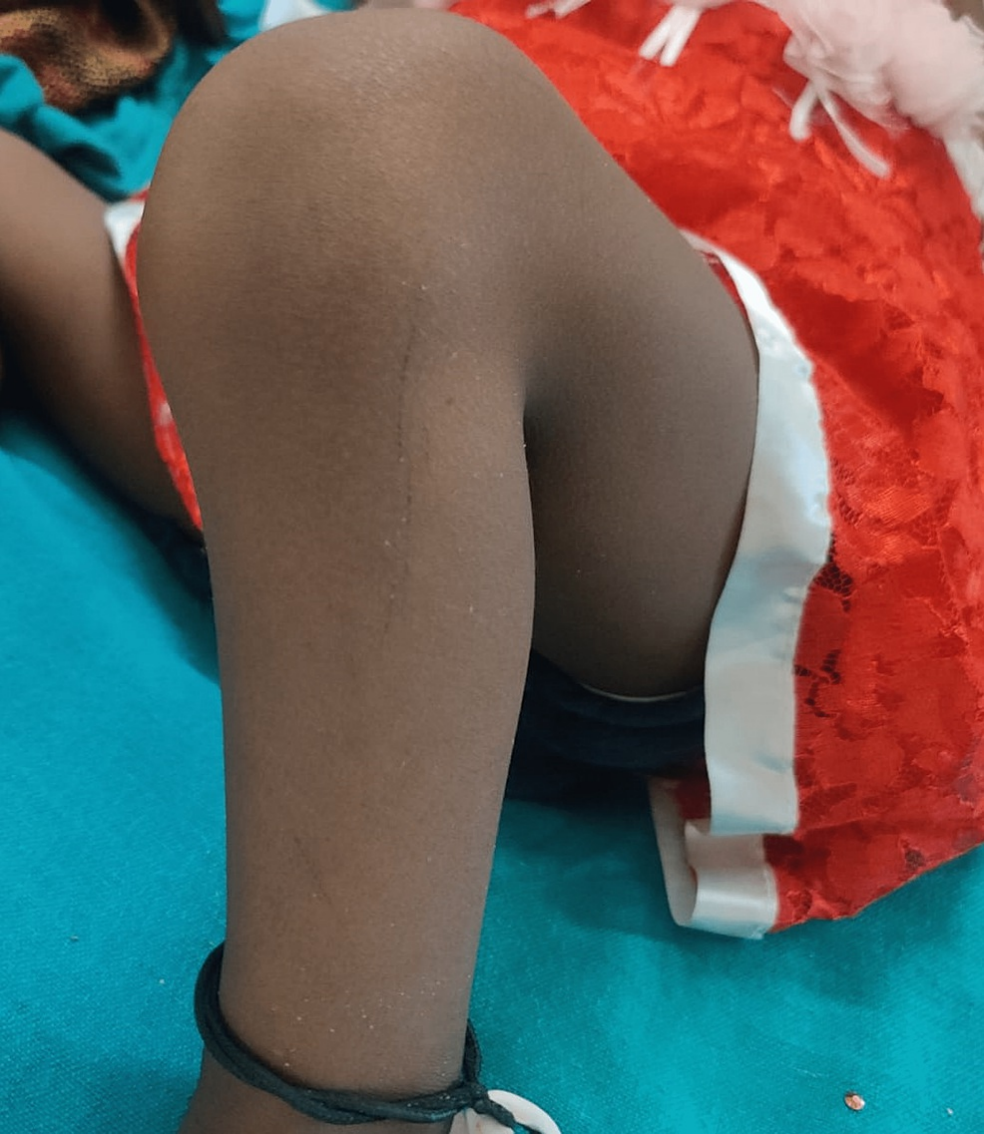
Showing chondrodysplasia

**Figure 5:**
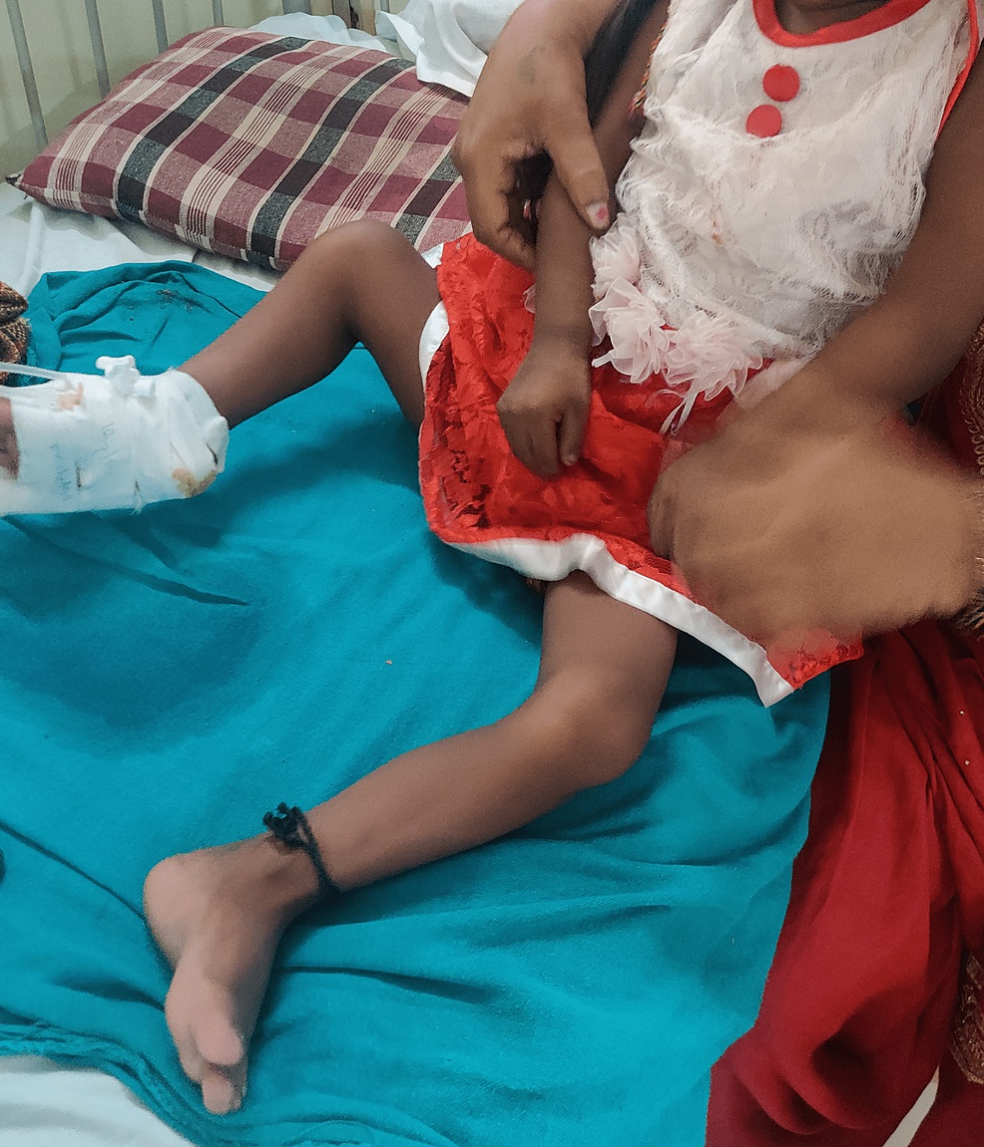
Showing bent lower limbs

**Figure 6:**
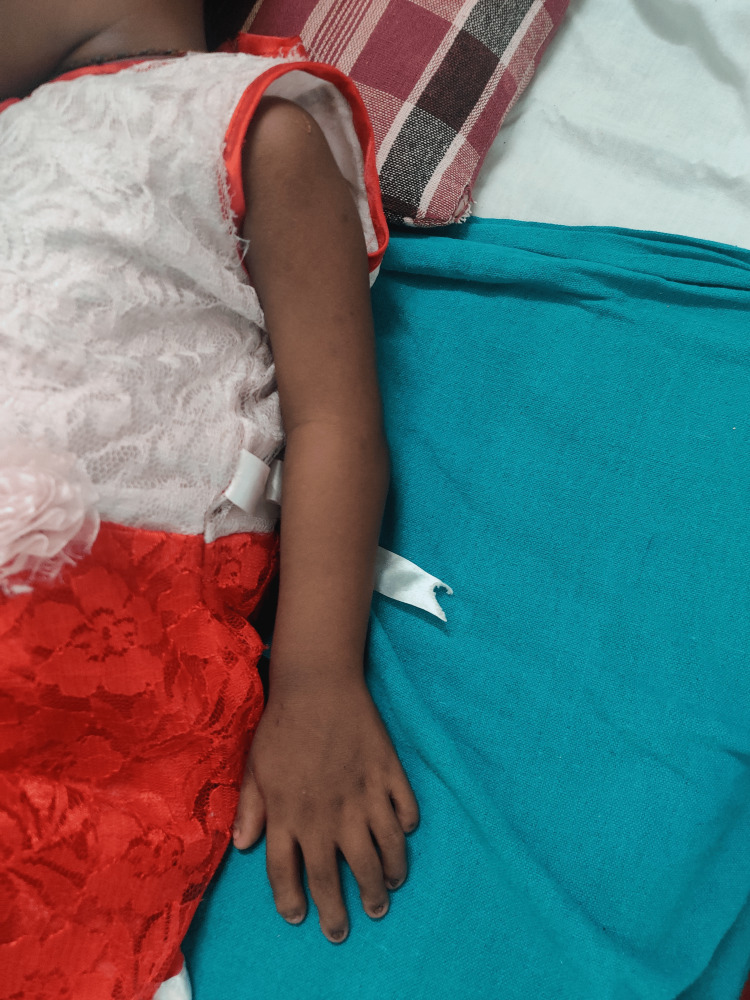
Showing deformities of limbs (short upper limbs)

**Figure 7:**
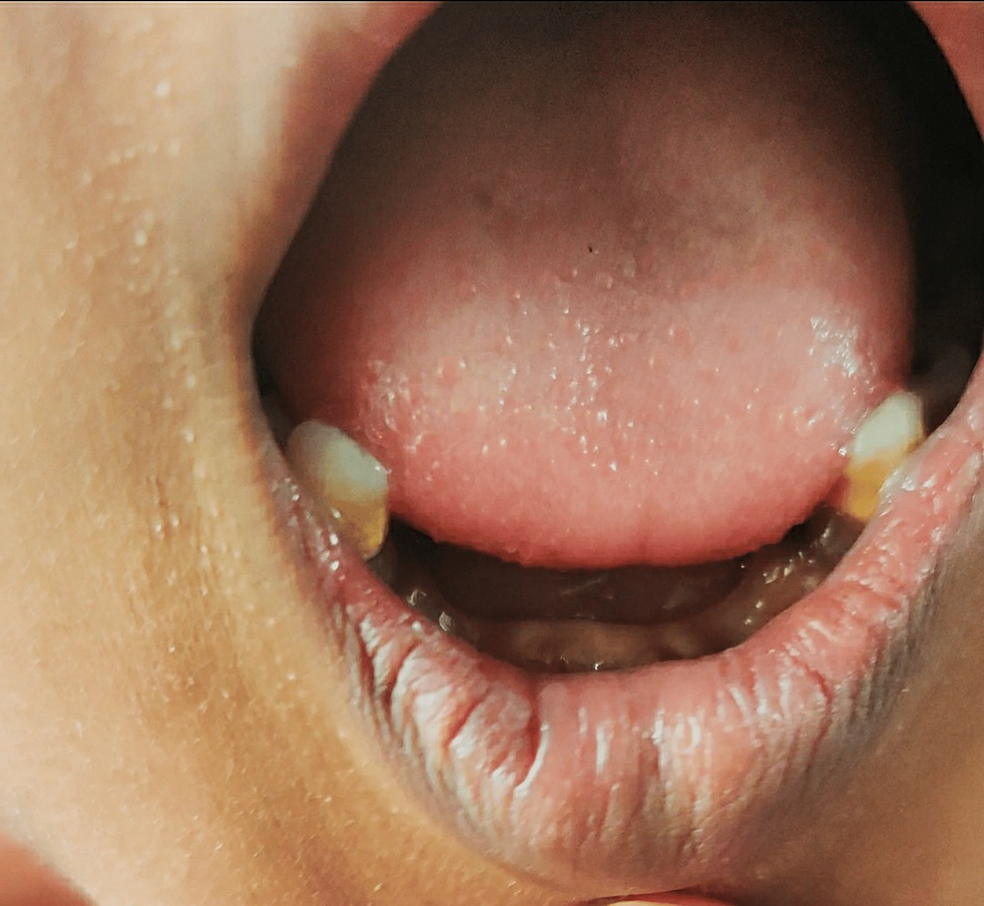
Oral cavity showing hypodontia

**Figure 8:**
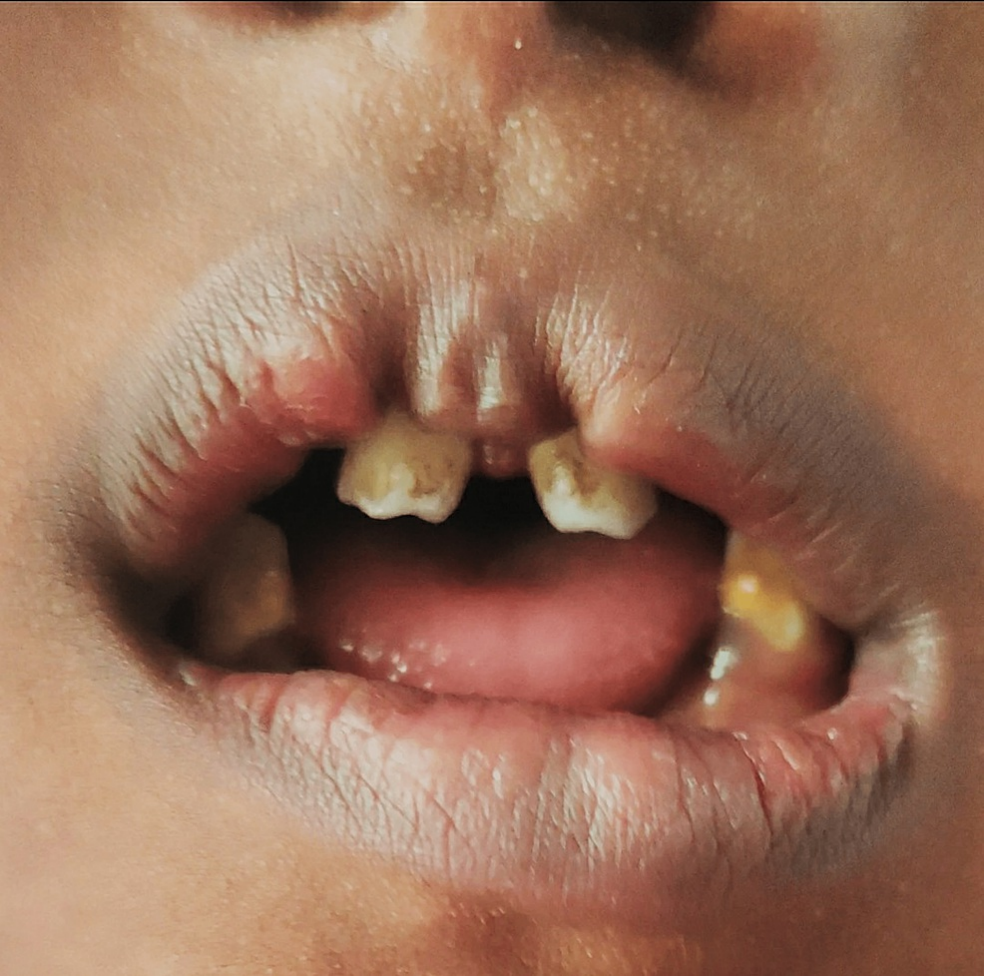
Oral cavity showing malocclusion

On general examination, the patient was conscious and well oriented with time, place, and person. Her heart rate was measured to be 124/min, and her respiratory rate was 46/min. She was afebrile. Blood pressure was 104/58 mm Hg, and pulse oximetry recorded oxygen saturation (SpO2) of 88% in room air. Cyanosis, clubbing, and oedema (pedal oedema) were present, whereas lymphadenopathy, pallor, and jaundice were absent. Other findings included polydactyly (presence of six digits in each of her hands; Figure 2), fingernail dysplasia (abnormally shaped nails seen on both hands; Figure 3), and chondrodysplasia (bone and cartilaginous deformity are seen over her knees; Figure 4). The upper limbs were shorter than the lower limbs, the lower limbs were bent (Figure 5), and the forearms were shorter in proportion to the arms (Figure 6). There were several orofacial manifestations like hypodontia (developmental absence of teeth in a few areas of gum; Figure 7) and malocclusion (upper and lower teeth were abnormally aligned; Figure 8).

A clinical examination revealed normal findings of the central nervous system (as per Glasgow coma score and reflex examinations), gastrointestinal system, and renal system. Respiratory examination revealed the presence of subcostal recession (SCR+), and air entry bilaterally equal (AEBE). Upon cardiovascular examination, the precordial bulge was seen, but there was no presence of apex impulse, dilated veins, scars, or sinuses. On palpation, the presence of thrill was heard on the apex beat over the fifth intercostal space and anterior axillary line. On auscultation, an ejection systolic murmur with loud P2 was heard (grade 4). Laboratory findings revealed hyperkalaemia (serum k+-5.2), hypercalcemia (serum calcium-7.8 mg/dl), hypomagnesemia (serum magnesium-3.9 mEq/l), increased serum phosphorus 3.9 mg/dl, and serum creatinine 1.6 mg/dl, but haematological findings were normal. 

**Figure 9:**
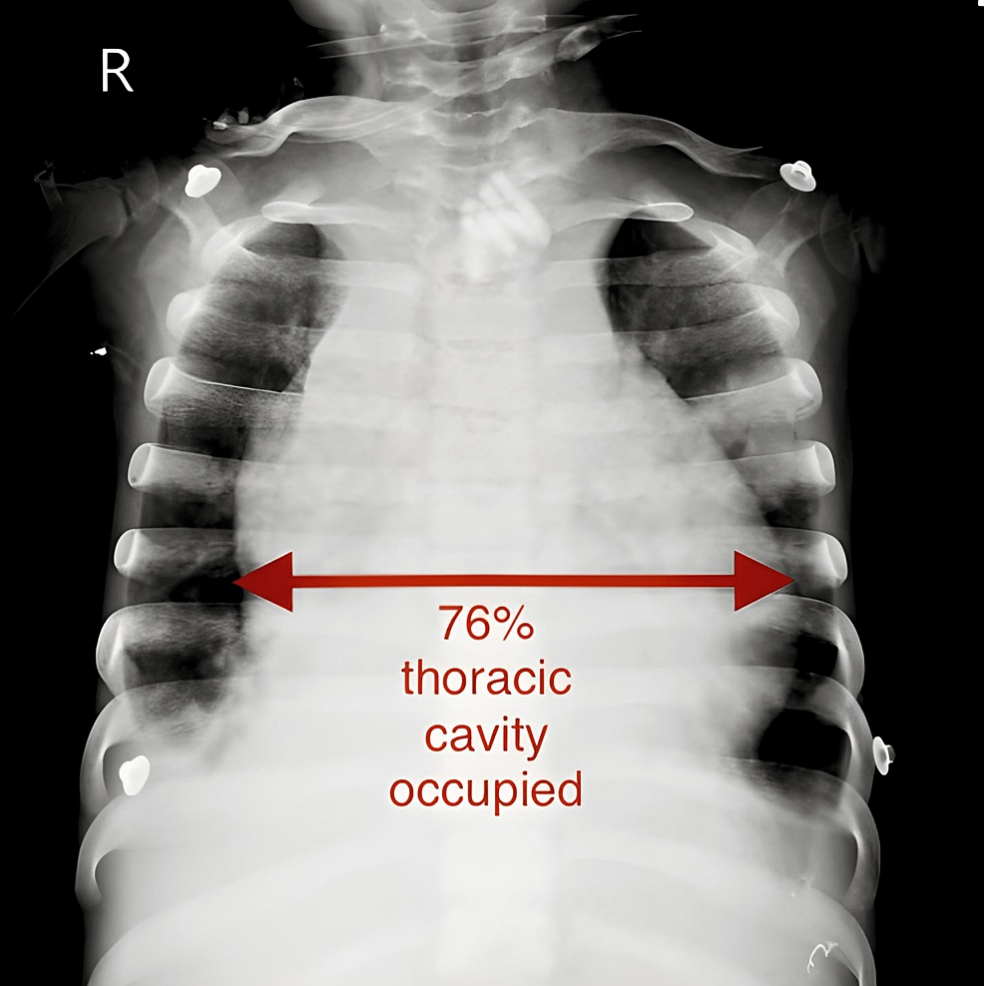
PA view of Chest X-ray (76% occupied by heart) PA=posterior-anterior

The respiratory system showed bilateral pleural effusion along with pericardial effusion. Arterial blood gas values were measured and showed 7.28 pH (reduced), pCO2 28.5 mmHg (reduced), and pO2 69.7 mmHg (reduced). PA (posterior-anterior) view of chest X-ray showed severe cardiomegaly and the heart is observed to occupy 76% of the thoracic cavity (Figure 9).

**Figure 10:**
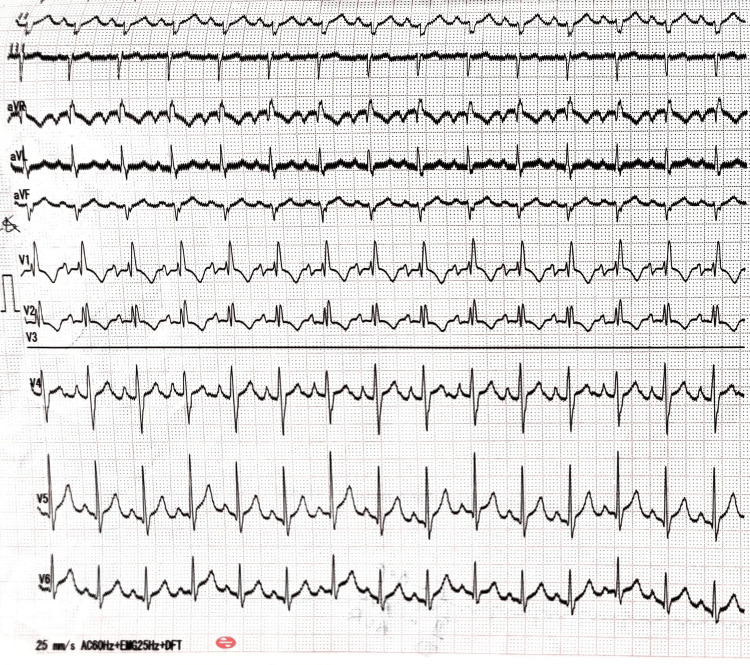
Showing ECG

**Figure 11:**
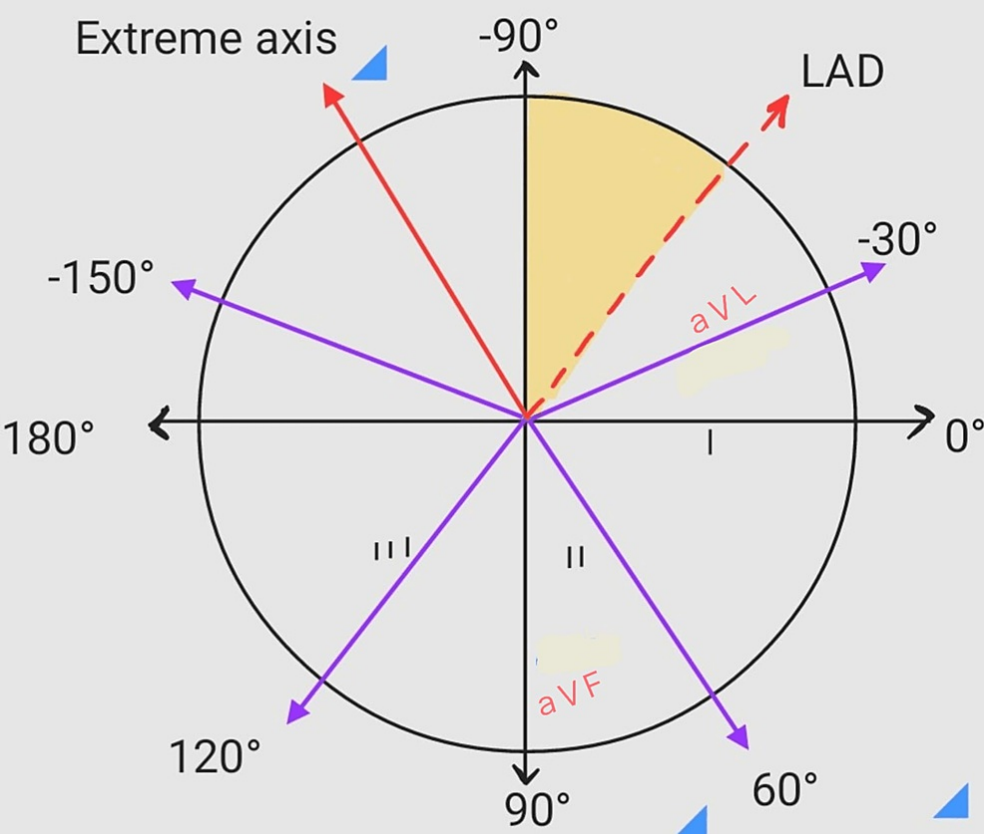
Depicting left axis deviation aVF=Vector Foot; aVL=augmented Vector Left; LAD=left axis deviation

In ECG, the PR interval was seen to be around 120 milliseconds and the QR interval was about 190 milliseconds (Figure 10). The ECG showed abnormalities in leads V1, V2, and aVR, T wave abnormality, and severity of left axis deviation (25mm/sec; Figure 11).

**Figure 12:**
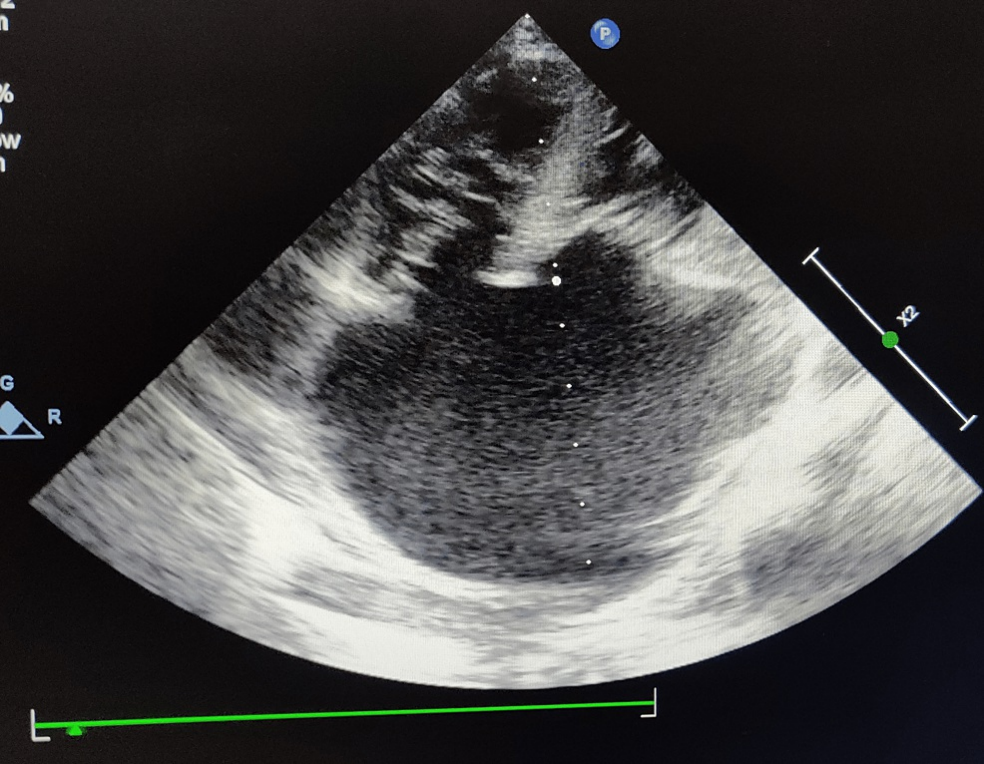
Common atrium and patent ductus arteriosus seen on echocardiography

Two-dimensional echocardiography (2D Echo) was performed on the patient, and it was concluded that the patient had levocardia and atrioventricular-ventriculoarterial (AV-VA) concordance. Three pulmonary veins were observed to be draining into the left atrium (left side), with no interatrial septum leading to the widening and formation of a common atrium, two equal ventricles. No shunt was detected in ventricular and great artery region, no coarctation of left aortic arch and, intact inferior vena cava. The ejection fraction was normal in the patient (Figure 12).

Several mentioned clinical features, pieces of evidence, and radiological findings directed that the patient was suffering from EVCS. 

## Discussion

EVCS is an autosomal recessive abnormality. As observed by Sharma et al., siblings had a risk of recurrence of 25% per offspring [11]. Patients are seen to have skeletal dysplasia. In the physeal growth zone, chondrocyte disorganisation in the cartilage of long bones was observed in foetuses with this syndrome. Variation in chondrocyte disorganisation was observed in the central physeal growth zone within the vertebrae [12]. EVCS is an infrequent disorder with a prevalence of seven per 1,000,000 cases [13]. Parental consanguinity is observed in around 30% of the total cases [14]. In our patient, parental consanguinity was present. A history of a similar younger sibling who died after one and a half months was stated by the patient’s mother. Neonatal history may be found to have small size after birth, retarded growth along with anomalies in the skeleton as the primary symptoms. The presence of natal teeth can be seen [12]. In our case, a positive neonatal history was found of normal weight after birth but skeletal anomalies and delayed developmental growth; the upper limb was shorter than the lower limb and the longer arm shorter than the forearm. Natal's teeth were absent. Congenital heart defects (CHDs) are seen to occur in some 50% of total cases. Atrial septal defect (ASD) is seen to be the most common condition and others include ventricular septal defect (VSD) and hypoplasia of the aorta [11]. It has been observed that most patients suffering from EVCS have mental retardation, and abnormalities in the central nervous system (CNS) like Dandy-Walker malformation (DWM) were reported in some cases [15]. Our patient presented with a normal IQ.

At the beginning of 4.5 months of gestation (18th week), USG (ultrasonography) and clinical examinations can help diagnose this syndrome [3]. One important and most common clinical finding observed in this syndrome is chondrodystrophy. In this case, most commonly, long bones of extremities like the femur (sometimes patella) are involved giving rise to a defect in ossification [1]. We usually observe that the thorax is sunken with funnel chest (pectus excavatum), inward curving of the lower back (lordosis), and inward turning of the lower extremities (genu valgum). Hairs are usually less and fine. The appearance of multiple digits (polydactyly), broad hands and feet, dysplasia of nails of fingers, and sausage-like fingers typically point towards the diagnosis of EVCS being very particular features for this syndrome [1]. Fissured tongue and cases of ankyloglossia are also seen [9,16]. Ruiz-Perez and Goodship stated in their work that the anomalies in EVCS are an outcome of the tissue-specific disruption of the reaction to Hedgehog (Hh) ligands [17]. Around 50-60 % of patients present with the features of congenital heart diseases (it could be septal defects, patency of ductus arteriosus, pulmonary hypertension, or valvular defects) in this case. All these cardiac anomalies lead to a decreased life expectancy in these patients [1,3,16]. Twenty percent of the cases present with genitourinary abnormalities, this could be agenesis or dysplasia of kidneys or renal system, or ectasia of the ureters and kidney stones [18]. Various abnormalities related to haematology have come to light. Dyserythropoiesis is described in one literature and perinatal myeloblastic leukaemia in another [3,19]. Congenital stridor can present as a rare disorder in a patient due to the emergence of a cyst that obstructs autonomous breathing in the upper airway by pushing the larynx, as reported by Digoy et al. [18]. Other uncommon features are epi- and hypospadias, cryptorchidism, strabismus, and malformations in the walls of both lungs or thoracic [3].

The beneficial and functional management of this syndrome should consist of a team comprising of a geneticist, oral and maxillofacial surgeon, pedodontist, prosthodontist, paediatrician, pediatric cardiologist, urologist, orthopaedician, psychologist, pulmonologist and pediatric neurologist [20].

## Conclusions

EVCS is very rare and still an incurable condition but there could be several kinds of management for this syndrome. The presence of several rare cardiac anomalies like patent ductus arteriosus (PDA), ASD, and three pulmonary veins in our case is rare in association with this syndrome. This condition can be surgically tackled in one sitting using an autologous pericardial patch along with ligation of PDA and ASD patch closure which will improve the condition and lifestyle of the patient. The extrapulmonary vein can be ligated. This case report will help readers gain knowledge about the rare findings of this particular syndrome and help medical professionals prognose, diagnose, and treat patients in an effective and faster manner.
